# Developing Spanish Language Intervention Content to Address Health Disparities among People Who Use Drugs, and Other Hard-To-Reach Populations

**DOI:** 10.1007/s11524-025-01050-2

**Published:** 2026-02-07

**Authors:** Ian David Aronson, Anthony Cramer, Robert Quiles, Olivia Marcus, Brent Gibson, Brittney Vargas-Estrella, Synn Stern, Chunki Fong, Alex S. Bennett

**Affiliations:** 1 NDRI-USA, 31 W 34 Street, Suite 8042, New York, NY 10001 USA; 2 OnPoint NYC, 104 -106 E 126 Street, New York, NY 10035 USA; 3 NYU School of Global Public Health, 708 Broadway, New York, NY 10003 USA

**Keywords:** Spanish, Language, COVID, Substance use, Drugs, Technology, Intervention

## Abstract

Our team developed content in English and Spanish for a technology-based intervention designed to increase vaccination against COVID-19 among people who inject drugs. From 2022 through 2024, we recruited 545 participants via peer referral, all of whom reported past 90-day injection drug use. Participants completed a tablet-based intervention, and software asked if they would like to vaccinate against COVID. Participants who did not vaccinate at the first visit received follow-up text messages. Participants who completed the intervention in Spanish were more than 2.5 times as likely to vaccinate at first visit (OR 2.525, 95% CI [1.059–6.076], *p* = 0.039), and 57% more likely to vaccinate at follow-up (OR 1.576, 95% CI [0.441–5.630], *p* = 0.484) compared to those who completed in English. These findings highlight the importance of developing intervention materials in languages beyond English. Research is warranted to understand how developing content in other languages may influence health outcomes.

## Introduction

People who inject drugs frequently lack access to care and may be reluctant to engage with providers because they fear stigma, disrespect, or mistreatment related to their substance use [1]. Only 14.6% of people diagnosed with a substance use disorder (SUD) received treatment in 2023 [2]. Various health services are available specifically for people who use drugs (PWUD), especially in New York City [3] where our research was conducted. Unfortunately, many of those most in need remain underserved due to a lack of effective outreach and basic communication barriers [4].

The above issues disproportionately affect people with limited English proficiency (LEP), which has been identified as a key social determinant of health and barrier to health [5, 6]. Low health literacy and LEP among Latinos, in particular, contribute to reduced quality of care, poorer healthcare outcomes, lower satisfaction with healthcare services, and greater mistrust toward healthcare providers [7, 8]. In a national survey of 6.8 million Latinos who were diagnosed with SUD but did not receive treatment, nearly all (97.1%) perceived that they did not need treatment [9], which suggests a significant gap in public health messaging toward LEP Spanish speakers, and/or problems with messaging effectiveness.

In New York, cultural and linguistic diversity creates important opportunities to explore new evidence-based intervention strategies, especially using technology. Technology-based interventions offer potential solutions because they can be made available in multiple languages with culturally appropriate content targeted to different groups. These interventions can then be quickly and efficiently delivered to a broad range of people in clinical and non-clinical settings. For example, interventions can deliver text messages or other content by mobile phone to facilitate continued engagement between (or perhaps instead of) office visits. This may particularly benefit people who speak languages other than English, yet must navigate an English-language healthcare system.

A literature search by our team did not find any systematic or scoping reviews of technology-based interventions created for non-English speaking PWUD in the United States, and found only a handful of randomized controlled trials for behavioral health interventions conducted in Spanish inside the US (e.g., [10, 11]). One study focused on racial/ethnic differences in substance use and harm reduction but “PWUD who spoke only Spanish were excluded from participation” [12].

Our team collaborated with bilingual peer educators and community members in New York City to develop a technology-based intervention designed to increase vaccination against COVID-19 among people who inject drugs. Using an iterative process, we first conducted qualitative interviews in English and Spanish to identify barriers to vaccination among people who inject drugs. We then created intervention videos in English and Spanish to address these barriers via messages created specifically for the target population. We delivered key intervention content by video because it does not require reading, and therefore, can be readily understood by LEP and other low-literacy populations. For more detail on our intervention and the content development process, please see Aronson (2022) [13].

Rather than developing our content first in English, and then translating into Spanish, we asked community members to appear on camera and address key points of information first in Spanish and then again in English, with the goal of creating linguistically and culturally centered materials [10]. We then evaluated the materials in a large-scale randomized controlled trial (RCT), conducted in partnership with a community-based harm reduction organization. The full results are discussed in a previous publication [14].

As part of the larger study, we conducted additional analyses of our RCT data to compare post-intervention vaccination rates among participants who completed the intervention in Spanish versus those who completed the intervention in English. We also examined what language participants reported speaking at home most frequently, what language participants chose for the intervention, and how these may have influenced vaccination rates.

## Method and Sample

From June of 2022 through October of 2024, we recruited a total of 545 participants via peer referral. Participants were aged 18 and older and reported injection drug use in the past 90 days. All intervention and informed consent materials were available in English and Spanish. Participants completed a tablet-based intervention that collected basic demographic information (age, gender, race/ethnicity) then measured knowledge of, and attitudes toward, COVID vaccination. The tablets also displayed a short (<6 min) educational video.

At the end of the intervention, software asked participants if they would like to vaccinate against COVID-19. Those who accepted the offer were vaccinated onsite by a trained nurse. Those who declined received automated text messages inviting them to return for follow-up. Participants had the option to return for up to two follow-up visits.

Our software separately recorded which language participants used to complete the intervention, whether participants self-identified as “Hispanic or Latino” or “Not Hispanic or Latino,” and also recorded participants’ response to the question “What language do you speak at home most frequently?” A total of 232 participants (42.6%) self-identified as Hispanic or Latino. Of these participants, 191 (82.3%) completed the intervention in English, and 41 (17.7%) completed the intervention in Spanish. Among participants who identified as Hispanic or Latino and completed the intervention in English, 146 (76.4%) reported they most frequently spoke English at home while 44 (23.0%) reported they most frequently spoke Spanish. Among those who identified as Hispanic or Latino and completed the intervention in Spanish, 39 (95.1%) reported they most frequently spoke Spanish at home while 2 (4.9%) reported they most frequently spoke English.

## Results

Participants were significantly more likely to vaccinate at the first visit if they completed the intervention in Spanish compared to English. Among people who completed the intervention in Spanish, 7 out of 41 (17.1%) vaccinated at the first visit compared to 38 out of 504 (7.5%) who completed in English (OR 2.525, 95% CI [1.049–6.076], *p* = 0.039). A similar, yet non-significant pattern emerged for people who vaccinated at follow-up: Among participants who completed the intervention in Spanish, 3 out of 27 (11.1%) vaccinated at a follow-up visit compared to 9 out of 110 (8.2%) who completed in English (OR 1.576, 95% CI [0.441–5.630], *p* = 0.484).

## Discussion

The findings that participants were more than twice as likely to vaccinate at first visit after completing the intervention in Spanish, and 57% more likely to vaccinate at follow-up, may hold great practical significance. Some cognitive studies suggest speaking in native vs. non-native languages can alter decision-making processes related to health care [5, 15], which in our study might have helped participants understand the importance and relevance of our messaging. Participants may also have responded better on an emotional level because they were viewing materials that depicted, and were specifically made for, native Spanish speakers. Our intervention may also have been the first time participants saw a thorough Spanish-language presentation explaining the importance of vaccination against COVID-19.

## Conclusion

Research is warranted to understand how intervention materials presented in multiple languages, and enabling participants to select a preferred intervention language, may influence health outcomes. Overall, these findings underscore the importance of developing intervention materials in multiple languages, and bring attention to the lack of behavioral health technology in languages other than English. It is also worth noting that 23% of participants who identified as Hispanic or Latino and elected to complete the intervention in English reported that they most frequently spoke Spanish at home. Further research is therefore warranted to examine why people choose one language over another, and how this may influence health outcomes.

## References

[1] Biancarelli DL, et al. Strategies used by people who inject drugs to avoid stigma in healthcare settings. *Drug Alcohol Depend*. 2019;198:80–6. 10.1016/j.drugalcdep.2019.01.037 PMC6521691 30884432

[2] Substance Abuse and Mental Health Services Administration. Key substance use and mental health indicators in the United States: Results from the 2023 National Survey on Drug Use and Health (HHS Publication No. PEP24-07-021, NSDUH Series H-59). *Center for Behavioral Health Statistics and Quality, Substance Abuse and Mental Health Services Administration*. 2024. https://www.samhsa.gov/data/report/2023-nsduh-annual-national-report.

[3] Jordan AE, et al. Drug overdose death following substance use disorder treatment termination in New York City: a retrospective longitudinal cohort study. *J Urban Health*. 2024;101(5):1045–57. 10.1007/s11524-024-00893-5 PMC11461374 39095494

[4] Khan GK, et al. Integration of a community-based harm reduction program into a safety net hospital: a qualitative study. *Harm Reduct J*. 2022;19(1):35. 10.1186/s12954-022-00622-8 PMC9002225 35414072

[5] Chipman SA, Meagher K, Barwise AK. A public health ethics framework for populations with limited English proficiency. *Am J Bioeth*. 2024;24(11):50–65. 10.1080/15265161.2023.2224263 37379053

[6] Sentell, T. and K.L. Braun. Low health literacy, limited English proficiency, and health status in Asians, Latinos, and other racial/ethnic groups in California. *J Health Commun.* 2012; 17 Suppl 3(Suppl 3):82–99. 10.1080/10810730.2012.712621 PMC3552496 23030563

[7] Hernandez J, et al. A systematic review and narrative synthesis of health literacy interventions among Spanish speaking populations in the United States. *BMC Public Health*. 2024;24(1):1–49. 10.1186/s12889-024-19166-6 PMC11210103 38926697

[8] Escobedo LE, Cervantes L, Havranek E. Barriers in healthcare for Latinx patients with limited English proficiency-a narrative review. *J Gen Intern Med*. 2023;38(5):1264–71. 10.1007/s11606-022-07995-3 PMC9888733 36720766

[9] Substance Abuse and Mental Health Services Administration. 2023 National survey on drug use and health: among the hispanic or Latino Population Aged 12 or Older. Substance Abuse and Mental Health Services Administration; 2024. https://www.samhsa.gov/data/sites/default/files/reports/rpt44472/2022-nsduh-popslides-hispanic.pdf. Accessed 22 Dec 2025.

[10] Olaya F, et al. A randomized controlled trial of the dissemination of an mHealth intervention for improving health outcomes: the WiseApp for Spanish-speakers living with HIV study protocol. *BMC Public Health*. 2024;24(1):201. 10.1186/s12889-023-17538-y PMC10792787 38233908

[11] Aguilera A, et al. Effectiveness of a digital health intervention leveraging reinforcement learning: results from the Diabetes and Mental Health Adaptive Notification Tracking and Evaluation (DIAMANTE) randomized clinical trial. *J Med Internet Res*. 2024;26:1–11. 10.2196/60834 PMC11496924 39378080

[12] Parker S, et al. Differences by race and ethnicity in drug use patterns, harm reduction practices and barriers to treatment among people who use drugs in Rhode Island. *Harm Reduct J*. 2025;22(1):38. 10.1186/s12954-025-01191-2 PMC11924686 40114268

[13] Aronson ID, et al. Using the participatory education and research into lived experience (PEARLE) methodology to localize content and target specific populations. *Front Digit Health*. 2022. 10.3389/fdgth.2022.992519. 10.3389/fdgth.2022.992519 PMC9634163 36339513

[14] Aronson ID, Quiles R, Cramer A, Fong C, Gibson B, Vargas-Estrella B, Stern S, Bennett AS. Designing and evaluating a technology-based intervention to address deep ambivalence about COVID vaccination among people who inject drugs. *Health Educ Res*. 2025. 10.1093/her/cyaf052. 10.1093/her/cyaf052 PMC12642696 41284226

[15] Białek M. The bilingual patient’s dilemma: same question, different answer. *Am J Bioeth*. 2024;24(11):84–6. 10.1080/15265161.2024.2399850 39401727

